# Creativity and REsilience Through Arts, Technology and Emotions: A Pilot Study on the Feasibility and Validity of the CREATE Platform

**DOI:** 10.3390/brainsci15111171

**Published:** 2025-10-30

**Authors:** Aristea I. Ladas, Christina Katsoridou, Triantafyllos Gravalas, Manousos A. Klados, Aikaterini S. Stravoravdi, Nikoleta Tsompanidou, Athina Fragkedaki, Evangeli Bista, Theodora Chorafa, Katarina Petrovic, Pinelopi Vlotinou, Anna Tsiakiri, Georgios Papazisis, Christos A. Frantzidis

**Affiliations:** 1 Department of Psychology, CITY College, University of York Europe Campus, 54626 Thessaloniki, Greece; arladas@york.citycollege.eu (A.I.L.); chrkatsoridou@seerc.org (C.K.); 3tgravalas@gmail.com (T.G.); mklados@york.citycollege.eu (M.A.K.); ntsompanidou@york.citycollege.eu (N.T.); tchorafa@york.citycollege.eu (T.C.); kpetrovic@york.citycollege.eu (K.P.); 2 Clinical Research Unit, School of Medicine, Aristotle University of Thessaloniki, 55134 Thessaloniki, Greece; kate_st@outlook.com (A.S.S.); georgepapaz@gmail.com (G.P.); 3 Cancer Patient Association (Kapa3), 11528 Athens, Greece; athinafraged@gmail.com (A.F.); libista@yahoo.gr (E.B.); 4 Occupational Therapy Department, University of West Attica, 12243 Athens, Greece; pvlotinou@uniwa.gr; 5 Neurology Department, Democritus University of Thrace, 68131 Alexandroupoli, Greece; anniw_3@hotmail.com; 6 School of Engineering and Physical Sciences, University of Lincoln, Lincoln LN6 7TS, UK

**Keywords:** aesthetic experience, anxiety, cognitive training, creativity, depression, digital mental health, emotion regulation, sentiment analysis, sleep quality, spontaneous eye blink rate, working memory

## Abstract

**Background/Objectives**: Anxiety and depression are prevalent global health concerns, especially prominent in vulnerable groups such as older adults, individuals with chronic health conditions (e.g., neurodegeneration and cancer), and those from low socioeconomic backgrounds. Digital interventions, including computerized cognitive training (CCT), show promise in addressing emotional dysfunctions in a more accessible and cost-effective manner. The CREATE platform aims to enhance Emotion Regulation (ER) through targeted Working Memory (WM) training, aesthetic engagement, and creativity, while accounting for dopamine activity via spontaneous Eye Blink Rate (sEBR). The purpose of the present study is to evaluate the platform’s feasibility and validity through a single pilot trial. **Methods**: The study enrolled twenty-seven healthy adults (aged 21–44) who completed standardized self-report questionnaires on sleep quality and ER. They were also enrolled in sEBR recordings and performed a CCT-adapted Corsi block-tapping task and an aesthetic art evaluation. Affective textual narratives and valence/arousal ratings were also collected. Participants were divided into “Good Sleepers” and “Poor Sleepers”. The platform evaluation enrolled a multi-modal pipeline including correlations and regression analysis of intervention metrics, sentiment analysis, and group comparisons. **Results**: WM task performance correlated positively with global ER and Cognitive Reappraisal scores. Post-training sEBR was significantly associated with ER, and lower sleep efficiency negatively impacted changes in dopamine activity (sEBR Diff). Dopamine activity of “Good Sleepers”, as indicated by sEBR, reached the high levels of the “Poor Sleepers” group after the training, suggesting a dopamine boost caused by the CREATE platform for those with quality sleep. Creativity and emotional expression, as indicated by sentiment analysis, were related to sleep quality. **Conclusions**: The CREATE platform shows promise in enhancing ER through multi-modal digital engagement, integrating cognitive training, art, and creativity. The findings support the inclusion of sleep and dopamine markers in intervention evaluation. Further studies with larger samples and clinical cohorts are warranted to establish efficacy and generalizability, as the present one was not powered to test the effectiveness of our training platform but was designed to assess its feasibility and validity instead.

## 1. Introduction

Generalized Anxiety Disorder and Major Depressive Disorder are considered leading mental health causes of disability and premature mortality, with a heavy financial burden [1,2]. More importantly, the prevalence of depression and anxiety seems to have spread around the globe, making no exceptions between high- and low-socio-demographic index countries [2], and is expected to increase [3]. The COVID-19 pandemic inflated the prevalence of mental health issues, with a particular impact on young adults: in 2019, the number of adults in many European countries with depressive symptoms doubled, and a four-fold increase was shown in young adults [4]. This phenomenon has been exacerbated by so-called COVID-19 pandemic era stressors [1]. For example, in a systematic review and meta-analysis of anxiety and depression rates in college students [5], increased reported cases of these mental health issues were found specifically in studies conducted after the pandemic. Adults with post-COVID-19 syndrome also show a considerable mental health burden due to increased depression and anxiety [6] or to increases in sleep issues, depression, and anxiety (for a systematic review and meta-analysis, see [7]). Individuals with chronic health issues, such as chronic pain patients, show even larger prevalence rates of anxiety and depression than healthy controls, approaching almost 40% (for a systematic review and meta-analysis, see [8]). This is further supported by studies on anxiety and depression in cancer patients (for a review, see [9]). Therefore, there is an urgent need for effective and sustainable mental health solutions.

Despite the high demand for mental health services, accessing them remains significantly restricted (for a systematic review, see [10]). For example, about two-thirds of mental health patients cannot access health services in the US at all, whereas the remaining one-third can access only basic care [10]. Regarding Europe, the EU Compass for Action on Mental Health and Well-Being 2016 survey indicated that the main barriers to accessing mental health care were the financial cost and the low availability of professionals. Overall, access to mental health care services is inadequate in European countries, and especially with regard to common psychiatric conditions such as depression [11]. Among mental health services in the European Union, it is psychosocial and psychological therapies that are insufficiently covered by member states [11].

Digital health solutions seem to be a promising alternative to traditional mental health care delivery modes, characterized by increased accessibility and low cost for the patient (for a review, see [12]). Internet-based mental health services can include structured assessments, screening, guided self-help, and expert-led treatments; specifically, regarding depression and anxiety, the most common and effective digital interventions have been found to be therapist-guided, followed by stand-alone Cognitive Behavioral Therapy (CBT), but also, non-CBT therapist-assisted interventions sometimes have modest effects (for a review, see [13]). Caution is warranted, though, when comparing effect sizes across studies due to the high variability in participant characteristics, as well as intervention lengths and types (for a review, see [13]). Computerized cognitive training (CCT) has also been shown to effectively reduce depressive symptomatology (e.g., [14]; for a review, see [15]), both in the cognitive and the affective domains in older adults with depression [16,17]. The higher accessibility and cost-effectiveness of CCT compared to traditional psychotherapy, combined with adherence monitoring, ability to tailor it to the participant’s skills, and repeatability, are characteristics that make CCT a promising alternative form of intervention [16]. Similarly, CCT has been used for anxiety reduction (see [18] for a review). For example, in one study, computerized training of Working Memory (WM) seemed to benefit the regulation of emotions, which in turn reduced anxiety levels [19]. More recently, WM CCT reduced social-anxiety-related outcomes following exposure to the feared context [20]. Overall, the limited number of studies employing WM and/or attentional control CCT to reduce anxiety or depression show some promising beneficial effects (see [18] for a review).

Encouraging as these may sound, the limitations of digital health solutions should also be considered. For example, despite the increases in patient accessibility, minority groups are still rather neglected in relevant studies. Moreover, usability and users’ needs must be investigated further (for a review, see [12]), as the sustained use and, therefore, effectiveness of these interventions largely depend on their ease of use.

Among other factors that may explain the limited effectiveness of these interventions, such as training duration, content, and adherence [21], the specificity of the training is considered quite important. That is, there is evidence to support focusing on one, instead of many, cognitive function results in more robust training effects, as it is more tailored to the impairment it addresses (for a meta-analysis, see [22]); it is more intensive instead of diluting the effort in several functions [23]; and it can lead to stronger transference of the training effect in related cognitive functions [24] and to increased adherence as users are enabled to observe their progress and, therefore, motivated to engage for longer [25].

Therefore, in our unique training e-platform, we targeted a single cognitive ability that plays a significant role in people’s well-being, Emotion Regulation (ER), an ability with established benefits for anxiety and depression symptomatology [26,27,28]. We hypothesized that populations vulnerable to these mental health conditions benefit the most from robust ER training. For example, recent evidence from cancer survivors supports the role of ER in moderating the relationship between cancer-related fatigue, anxiety, and depression [29]. Given the highly complex nature of ER, making it very difficult to design a training protocol to directly influence it, we chose to include evidence-based cognitive training tasks that tap into the very cognitive functions that support ER, such as WM. As aforementioned, CCT tapping into WM has been shown to reduce anxiety levels in people with relevant symptomatology [19,20]. In a similar vein, the role of WM and its interaction with ER in influencing mood disorder characteristics are stressed by findings showing increases in depressive and manic symptoms when WM is poor, as this in turn leads to poor ER ability [30]. More specifically, several ER strategies have been related to WM, such as reappraisal and suppression [31,32]. The suggested mechanism is that people with inefficient WM ability probably either misuse their ER or choose ER strategies that do not benefit them; this is not surprising, given the neurocognitive overlap between WM and ER [33]. On a neuronal level, studies show increased brain activity in prefrontal regions traditionally related to WM when ER strategies, such as reappraisal, are used [34]. In turn, reappraisal is particularly valuable to depressed individuals, as it is used to discourage negative thoughts and change them to different ones [35]. Similarly, for people with anxiety, reappraisal is considered the most effective ER strategy they can use [36]. Another path through which WM interacts with ER is through affective control (i.e., the ability to focus on the relevant affective information and inhibit the irrelevant ones; [37]): Emotion Regulation strategies are flexibly deployed through affective control, and ER strategy maintenance and monitoring, two core functions of WM, are necessary in this process [38]. Of note is the argument that striatal dopamine may be responsible for the significant improvements in ER through WM training in some studies, whereby dopamine activity (DA) in the striatum may have been increased due to a placebo expectation of experiencing cognitive improvements from training; in turn, striatal dopamine is heavily involved in the gating aspect of WM (for a review, see [39]). Such an argument speaks to the crucial role of DA in WM and its effects in ER; we, therefore, considered it imperative to assess DA and thus included the spontaneous Eye Blink Rate (sEBR) measurement in our platform, considered an established proxy of striatal dopamine activity [40]. An additional reason to include DA assessment was to further establish the validity of the CREATE measures in assessing sleep quality, ER, and WM: Firstly, DA plays an important role in sleep by regulating sleep states [41,42]; therefore, we would expect to see relationships between sEBR and sleep self-reported scores, suggesting that the measure we have included is a valid indicator of quality of sleep. Secondly, there is evidence to support striatal dopamine’s role in emotional processing ([43]; for a review, see [44]). This suggests a positive correlation between ER and sEBR that would underline the utility of the ER measure included in the CREATE platform. Thirdly, DA has been suggested to be the main neuromodulator of WM [45,46]; thus, if our game taps into WM, then participants’ game performance will correlate with sEBR.

Following the recent trend of using art (i.e., artistic creativity; aesthetic experience) as a means of promoting ER and wellness, we added aesthetic experience in our “toolkit”, which significantly contributes to ER and overall well-being (for a review, see [47]). In addition, the inherent connections between emotional processing and art, with artworks, for example, being described in terms of emotions elicited, made visual art stimuli an ideal candidate tool for the CREATE platform. The detailed mechanism through which art exposure influences ER is described in [47]. Moreover, there is an inherent link between aesthetic experience and WM: aesthetic experience is modulated by WM load, with a higher WM load decreasing aesthetic experience significantly [48]. Particularly with regard to visual art, studies suggest that aesthetic experience is modified by individual differences in WM [49,50]. Therefore, we considered that visual art stimuli would be suitable for our platform. We also added creativity through the production of art-related textual narratives, as it is considered a major facilitator of ER [51,52], given the shared neural underpinnings among these large-scale networks (for a review, see [53]). More specifically, to gauge our participants’ creativity, we used Expressive Writing (EW) (i.e., emotional expression through writing) by asking the participants to write a few sentences describing the emotions that each art stimulus elicited. Expressive Writing has been widely used as a psychological intervention tool for people with traumatic experiences [54]. It is important to note that EW has been traditionally found to reduce biological markers of stress and stress-related illness [55,56]. It has also been related to a long-term reduction in anxiety in healthy adults [57]. The more plausible explanation of this therapeutic effect of EW, according to the systematic review by [58], is that it functions as an effective strategy of ER by regulating affect through emotional expression [59].

Another unique characteristic of the CREATE platform is sleep quality assessment, as the causal role of quality sleep in ER has been well established (for a review, see [60]). Finally, we also measured the platform’s Affective Usability and the users’ experience by employing a CCT usability scale created by one of the main authors of this paper, initially designed for the purposes of a previous project funded by the European Commission, which aimed to promote active and healthy aging through computerized neuroplasticity-based, serious games [61].

Summing up, the primary objective of the present study was to test the feasibility (i.e., adherence; usability) and validity of the CREATE platform, not its effectiveness as an e-health intervention. Thus, according to the theoretical framework and the literature presented above, we formulated the following hypotheses:

**Hypothesis** **1.** *CREATE platform development: The creation of our training platform is based on the hypothesis that there is a positive triple association between WM performance, ER, and creative expression (EW and aesthetic evaluations as measured by sentiment indices).*

**Hypothesis** **2.** *The modulatory role of DA: We further hypothesize that DA (as measured by sEBR) will be significantly correlated with WM performance, ER scores, EW indices of verbal creativity and emotionality, and sleep quality measures.*

**Hypothesis** **3.** *The role of sleep quality in cognition and ER: Finally, we expect that sleep quality will be positively associated with both ER and creative expression (EW). No hypothesis was provided regarding the usability of the platform, as we wanted to stay as open as possible to the feedback of the participants, for improvement purposes.*

**Hypothesis** **4.** *Given the significant influence of WM in aesthetic experience, we expected that if our WM task indeed taps into WM and if our visual art stimuli elicit aesthetic experiences, then WM task scores will be related to aesthetic experience scores.*

## 2. Materials and Methods

**Figure 1:**
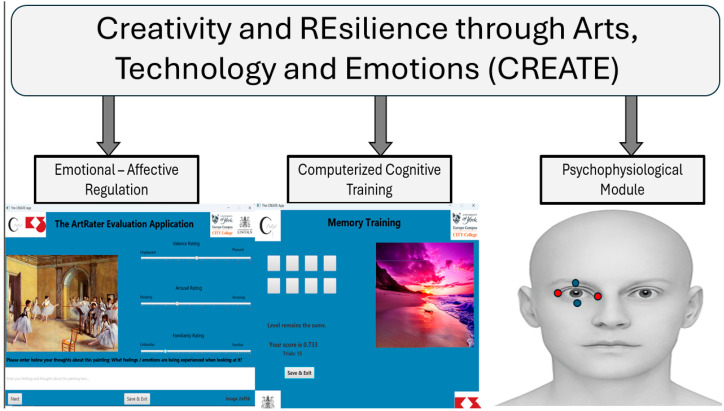
Overview of the CREATE platform structure and user interface. The upper panel illustrates the data collection workflow across three modules: the affective module (ArtRater Evaluation Application), the cognitive module (Memory Training tasks including Corsi block-tapping and N-back), and the psychophysiological module (sEBR recording). Arrows indicate the sequential flow and integration of data streams into a unified repository. The lower panel displays representative screenshots from the CREATE application: (**Left**) Memory Training interface showing adaptive task feedback, (**Middle**) Working Memory task with correct response feedback, and (**Right**) ArtRater Evaluation interface displaying emotional rating scales and narrative input fields.

CREATE (Creativity and REsilience through Arts, Technology, and Emotions) is a multi-domain platform used for computerized cognitive training (CCT), creativity elicitation, and assessment of mental and cognitive well-being (see Figure 1). It introduces a unified framework for assessing and later training in affective, cognitive, and psychophysiological measures. Currently, it comprises three interrelated modules:

(1) An emotional affective regulation module, which captures self-reported affect, perceived beneficence, and creativity through emotion-related evaluations and free-text narratives based on artistic stimuli;
(2) A computerized cognitive training suite to assess working memory and attentional flexibility through gamified tasks (e.g., Corsi block-tapping; N-back) of adaptive difficulty;
(3) A computer-vision, psychophysiological module for unobtrusively recording spontaneous Eye Blink Rate (sEBR) as an indirect index of dopaminergic activity. An agile methodology through living lab methodologies was used to elicit the functional and technical requirements that allow for the synchronous collection and integration of behavioral and physiological data, aiming to explore how emotional and cognitive processes interact in creative and resilient functioning.

### 2.1. Participants

**Figure 2:**
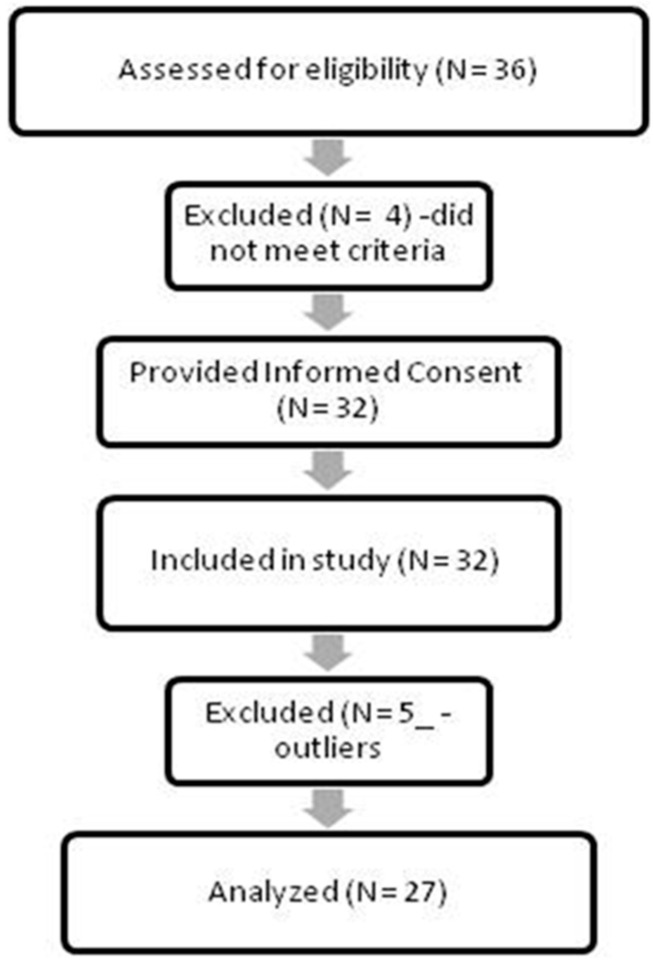
Flow diagram showing the number of subjects approached, consented, excluded, and analyzed.

For the purposes of this pilot study, we initially approached 36 individuals and recruited 32 adults, out of whom 5 were excluded as outliers in our exploratory analysis (see Figure 2). Therefore, 27 young and middle-aged adults (age range 21–44) were included, of both genders (15 women, 12 men), with a higher education level, with an object of study/employment related to cancer patients/survivors, of Greek origin, free of neurological and psychiatric diagnoses and medication, and fluent in the English language as self-reported. One participant was a cancer survivor. An informed consent form was signed prior to participation by all volunteers. The study was approved on 8 May 2024 by the Ethics Committee of the Psychology Department of CITY College, University of York Europe Campus (Ethics Application No 1063). As this was a pilot and feasibility study and not a randomized control trial, we used convenience sampling and pseudo-randomization of participants based on sleep quality post hoc.

### 2.2. Measures

#### 2.2.1. Affective Usability and User Experience Assessment

A self-report scale was used as the primary outcome measure of the usability of the CREATE platform. This consisted of 26 items, answered on Likert scales, with higher scores representing higher usability and acceptability. Six subscale scores can be derived (Computer Literacy, Affective Evaluation, Interface, Perceived Beneficence, User Engagement, Sustainability). Three open-ended questions are included at the end, asking participants to describe the perceived benefits and drawbacks of the platform, as well as to suggest what kinds of games they would like us to include. The questions were designed according to research on the usability and commercialization of human–computer interface systems [62,63]. For a detailed description of the scale, please see [61]. As a secondary measure, we took note of the participants’ verbal comments during and after they engaged with the platform.

#### 2.2.2. Psychological Self-Reported Scales Included in the CREATE Platform

The Pittsburgh Sleep Quality Index (PSQI [64]) was used to thoroughly assess sleep quality, as it is one of the most widely used self-report questionnaires in sleep medicine with strong psychometric properties (for a review and meta-analysis, see [65]). The PSQI includes 19 items separated into 7 subcategories, which represent different aspects of sleep quality: (1) Perceived Sleep Quality, (2) Sleep Duration, (3) Sleep Latency, (4) Sleep Efficiency, (5) Sleep Disturbances, (6) Daytime Dysfunction, and (7) Use of Sleep Medication. Global sleep quality was also derived, ranging from 0 to 21, with higher scores indicating worse sleep quality and a threshold of 5 demonstrating good sensitivity and specificity in distinguishing poor from good sleepers [64].

The Emotion Regulation Questionnaire (ERQ [66]) was employed to assess ER. It was designed to assess the ER strategies of Cognitive Reappraisal and of Expressive Suppression. It consists of 10 items assessed on a Likert scale, from 1 (strongly disagree) to 7 (strongly agree), with higher scores indicating better ER strategies.

#### 2.2.3. Computerized Cognitive Task Included in the CREATE Platform

We created an adaptation of the forward Corsi block-tapping neuropsychological test (CBT [67]), in a computerized format, to train visuospatial WM. Traditionally, the block span (i.e., the maximum number of blocks tapped correctly) is the score derived from this test, representing visuospatial WM capacity. Computerized versions of this test have also used the percentage or the total number of sequences correctly tapped (i.e., with the mouse censor) out of the total task trials (for a review, see [68]). However, in our version of the CBT, there are several scores that can be derived, allowing for a more integrative assessment of WM capacity (see Table 1 for a description of all scores). The visuospatial WM game application was developed using JavaFX 23 and was designed to assess and enhance the user’s WM through a progressively challenging visual recall task. The development methodology is rooted in core principles of cognitive training through adaptive difficulty according to the user’s performance.

The game’s objective was to test the user’s ability to memorize and recall a sequence of numbers displayed briefly on a set of buttons. Each game session involves a cycle of displaying randomized numbers. Then, those numbers disappear, and the user is prompted to reproduce the same sequence from memory by clicking the buttons in the correct order. The game incorporates an adaptive learning approach, adjusting its difficulty based on the user’s performance as follows:

- The difficulty level is evaluated after every 10 trials.
- If the user’s accuracy (score) exceeds a predefined threshold, the level increases, and the numbers are displayed for a shorter duration, making the task more challenging.
- If performance drops below a certain point, the display duration may increase to ease the difficulty.

Each round follows a trial–feedback loop. Initially, the sequence of numbers is briefly displayed, and then, the numbers are hidden. The user is prompted to recall and click the buttons in the correct order. Immediate feedback is provided, indicating whether the sequence was correct or not. The user’s score is constantly updated based on cumulative correct and total trials. This is achieved by using dynamically updated labels to display score, game level, and trial count. Aiming to further enhance user motivation through a reward approach, there are visual elements (image parts) that are progressively displayed after each correct answer, thus reflecting the user’s progress. This gradual visibility of image components based on the user’s performance acts as a form of visual reinforcement. The application performs user tracking and longitudinal evaluation of the user’s performance by logging key metrics (score, level, number of trials), along with timestamps, to a file. This supports review of performance over time and enables future analytics and adaptive learning insights.

The rationale of this dynamic adjustment is to maintain engagement and to ensure the game remains appropriately challenging for users of varying skill levels. From a software architecture perspective, the system is designed to be modular. The Graphical User Interface (GUI) components are separated using FXML for clarity and ease of modification. A logic-controlling adaptation of the Corsi block-tapping neuropsychological test [58], in a computerized format, was used to train visuospatial WM. The dependent variables are the number of correct responses, the mean and median correct responses, and the maximum number of blocks remembered correctly. The memory game application was developed using JavaFX and was designed to assess and enhance the user’s short-term memory through a progressively challenging visual recall task. The development methodology is rooted in core principles of cognitive training through adaptive difficulty according to the user’s performance.

#### 2.2.4. Aesthetic Experience Through Art Evaluation Included in the CREATE Platform

Aiming to quantify aesthetic experiences, we developed a JavaFX-based application acting as an art-rating tool. It was designed to gather users’ affective and cognitive responses to 15 images of famous paintings (visual stimuli). The system integrates subjective ratings, textual annotations, and interaction tracking to support research with applications in psychology, affective computing, and human–computer interaction. The main goal of the system is to present a random sequence of images on the computer screen and collect the user’s responses in terms of arousal (emotional intensity), valence (emotional pleasantness), familiarity (subjective sense of recognition), and sentiment (free-text reflection). These dimensions are widely recognized in psychological models of affect and are commonly used in studies involving the estimation of the emotional content from affective visual stimuli [69,70].

These art stimuli were carefully chosen by one of the authors (C.K.) with a specialty in both psychology and art due to their differing emotional valence and arousal elicited when viewed. Initially, a predefined collection of image paths is randomly shuffled, ensuring that the presentation order varies across sessions. This prevents ordering effects and supports unbiased data collection. After each art stimulus presentation, the user interacts with three sliders to input numerical ratings from 1 to 10 for arousal, valence, and familiarity. Additionally, a text field prompts the user to express the emotions or thoughts induced by the specific stimulus, thus capturing qualitative responses such as sentiment and interpretations.

Slider values are interactively adjustable via mouse input. The application provides a smooth interface for real-time input without cognitive overload. Upon rating each image, the system retrieves the user’s input values and logs them to a local file, along with the image identifier and timestamped sequence. The logging mechanism supports subsequent data analysis, including correlational studies, sentiment analysis, and predictive modeling of affective responses. Each user has a uniquely named file to track personalized data across sessions. The application is designed to maintain a sequential flow: upon pressing the **Next** button, the current image’s data is saved, and the next image is displayed. When all images have been saved, the session concludes automatically. The **Save** button allows for early exit while preserving the latest input, returning the user to the main menu for further actions or repeated tasks. A counter label informs the user of their progress (e.g., “Image **3 of 6**”), providing orientation and motivation. The sliders are initialized with default values to streamline interaction and reduce error potential.

There are six scores (dependent variables) that can be derived from the natural language processing of the textual narratives: **Polarity** is a numerical score that ranges from −1.0 to 1.0 and reflects the emotional tone of a participant’s comment. A Polarity score of −1.0 indicates a very negative sentiment (e.g., sadness, anger), a score of 0.0 reflects neutral sentiment, and a score of 1.0 represents a highly positive sentiment (e.g., happiness; admiration). This measure helps assess how emotionally positive or negative a comment is. We used the Polarity score as a sentiment and emotional writing indicator, with both negative and positive Polarity indicating more EW. **Subjectivity** measures the degree to which a comment is based on personal opinions, feelings, or beliefs and ranges from 0.0 to 1.0. A score close to 0.0 suggests the language is objective or fact-based, while a score near 1.0 implies the text is highly subjective or emotionally expressive. This helps gauge the level of personal engagement and emotional investment in the response. Similarly to the Polarity score, the Subjectivity variable was taken as an indication of emotionally loaded EW, with higher Subjectivity indicating more emotional expression. **Average Word Length** (AWL) represents the mean number of characters per word in a comment. While the typical range for English is around 4 to 7 characters, higher values may indicate the use of more complex or sophisticated vocabulary, while lower values could point to simpler, more colloquial language. Interestingly, in psycholinguistics, word-formation is argued to be deeply rooted in the person’s creative potential [71]; therefore, we considered the Average Word Length score as a useful indicator of users’ linguistic creativity. **Token Count** refers to the total number of words (tokens) used in a comment. While there is no fixed range, larger values indicate longer and potentially more detailed or expressive responses. This feature is useful for understanding how verbose a participant is in describing their impression of an image. **Unique Tokens** count the number of distinct words used in a comment. Like Token Count, it has no fixed range but depends on the length and richness of the response. A higher number of Unique Tokens often suggests a broader vocabulary and less repetitive language. **Type–Token Ratio (TTR)** is the ratio of unique words to total words in a comment and ranges from 0.0 to 1.0. A TTR close to 1.0 indicates that nearly every word is unique, reflecting high vocabulary diversity. In contrast, a lower TTR suggests more repetition and limited lexical variety. This is a widely used metric in linguistic analysis used to measure language richness. For the purposes of the present study, the first three scores (Polarity, Subjectivity, Average Word Length) were included in the analysis.

The CREATE platform was developed at the University of Lincoln by the senior author (C.A.F.) following an agile methodology involving relevant stakeholders from all the contributing authors and institutions. A living lab approach was used to require the system’s functional and technical requirements and to result in the present methodology, which is suitable for a variety of use cases, such as (1) experimental psychology studies measuring emotional responses to affective stimuli, (2) user experience (UX) research for evaluating emotional engagement with visual content, and (3) training datasets for machine learning models in affective computing.

#### 2.2.5. Assessment of DA with sEBR Included in the CREATE Platform

The senior author (C.A.F.) also created a Python 3.6-based Graphical User Interface (GUI) for quantifying spontaneous eye blinks in real time using a standard RGB webcam. The system is based on Google MediaPipe technology [72]. A Tkinter GUI provides immediate visual feedback to both the participant and the experimenter, displaying

- A live video stream with facial landmarks;
- A waveform of the eye-opening ratio;
- A real-time blink counter;
- A session timer.

This design enables non-invasive monitoring of blink activity, which can be used in a plethora of studies (behavioral, psychophysiological, fatigue). The system’s application combines computer vision (face and eye landmark tracking), signal processing (blink event detection), and human–machine interface methodologies. The GUI collects subject-specific metadata (name, age, and sex) and a user-defined blink threshold that controls the sensitivity of the computer vision detection system. The GUI is simple and minimal, consisting of two status labels displaying (1) the total blink count and (2) the elapsed duration of the recording session. A “**Start Recording**” button initiates real-time acquisition and analysis. When the session ends (user presses “**q**”), the results are automatically saved to a comma-separated value (.csv) file.

### 2.3. Procedure

The participants were asked to sit comfortably in a chair in front of a desk, with a laptop in front of them. The information sheet and the consent form were presented in digital form on the laptop, as well as the self-report scales, the WM task, the art evaluation tasks, and the sEBR recording. After the participants read the information sheet and signed the consent form, they were asked to complete the PSQI and the ERQ. The sEBR measurement followed. Next, half of the participants continued with the art evaluation task, while the other half engaged with the WM task. Task order was counterbalanced to minimize carry-over and order effects. Engagement with the platform lasted 30 min on average.

## 3. Results

### 3.1. Primary Analyses: Feasibility

#### 3.1.1. Affective Usability and User Experience Analysis

##### Quantitative Data

The means and standard deviations of all subscale scores are presented in Table 2. The maximum Likert scale score for the Computer Literacy subscale was 4; for the Affective Evaluation subscale, it was 7; and for Usability, User Engagement, and Perceived Beneficence, it was 5. For Interface, the maximum score was 2, and for Sustainability, it was 8. The higher the number, the more positive the evaluation. Overall, users reported high levels of usability, positive User Experiences, relatively high perceived benefits gained, and positive affective states related to the CREATE platform. With regard to adherence, all participants completed all the tasks of the CREATE platform (i.e., adherence rate, 100%).

##### Qualitative Data

We combined the answers from the two open-ended questions in the usability scale with the notes for the participants’ comments during and after their engagement with the platform. We then used the principles of Thematic Analysis to derive meaningful patterns, represented as “Themes”, from that data (please see Table 3). In terms of the beneficial aspects of the CREATE platform, users appreciated the interactivity, the gamified nature of the application, and the integration of technology. They found it fun and engaging, and according to their reports, these features kept their minds alert. It was described as user-friendly, easy to use, creative, and challenging. With regard to the platform’s drawbacks, users reported some issues with functionality and design, with requests for better platform layout and question arrangement. Some technical issues were also reported, such as sometimes randomly returning to the first difficulty level. Some users also requested increasing the number of squares and frames in the CCT to make it more challenging and engaging. Finally, when asked what kinds of games they would like us to include, users expressed interest in the development of memory games, especially with images, shapes, and colors, including games with image pairs or more varied visuals; a Stroop test-style game; and a game involving commenting on artworks to promote creativity.

### 3.2. Secondary Analyses: Validity

The examination of Hypothesis 2 was conducted with Permutation Correlations and independent *t*-tests. Assessments of Hypotheses 1 and 3 were conducted using Permutation Correlations and a regression model.

#### 3.2.1. Analyses Examining Hypotheses 1, 2, and 3: Sentiment Analyses, WM Task Performance, Sleep Quality, ER, and sEBR

Outliers were initially filtered out, with a filter of two SDs above/below the mean for each variable. From the WM task, we included three variables in the analysis: total number of correct responses, the maximum correct number of blocks selected, and mean correct responses; from the ER scale, we included the total ER score, and the two sub-scores of the ER strategies assessed (i.e., one total score for Cognitive Reappraisal and one total score for Expressive Suppression); from the PSQI scale, means from all sleep dimensions assessed were included (sleep latency, sleep subjective quality, sleep duration, sleep efficiency, sleep disturbances, and daytime dysfunction), along with the TotalPSQI score. Regarding DA, we included the mean sEBR pre- and the mean sEBR post-task engagement, and we also calculated the sEBR Diff (i.e., difference between pre- and post-sEBR, expressed as sEBR post–sEBR pre). From the Sentiment Analysis of the participants’ creative writing texts, we included all scores (i.e., Subjectivity, Polarity, Token Count, Unique Tokens, Type–Token Ratio, and AWL; see Table 4 for means and SDs of all variables).

##### Correlation with Permutations

Our exploratory statistics indicated a violation of the normality and the homogeneity of variance assumptions for some variables; therefore, we used a non-parametric test to assess relations among our variables. To investigate the relationships between the behavioral, blinking, sleep, sentiment, and ER variables, we applied a non-parametric, permutation-based correlation analysis. Data was imported into Python 3.6 and restricted to the predefined set of outcome variables of interest. Pairwise associations were estimated using Spearman’s rank-order correlations, which are robust to non-normal distributions and appropriate for ordinal or skewed variables.

To evaluate the statistical significance of the observed correlations, we implemented a permutation testing approach. Specifically, the values of each variable were randomly permuted across participants while preserving their original distribution, and Spearman correlations were recomputed for each permutation. This procedure was repeated 2000 times to generate an empirical null distribution of correlation coefficients for every variable pair. Empirical *p*-values were then calculated as the proportion of permuted correlations that were greater than or equal in magnitude to the observed correlation.

**Figure 3:**
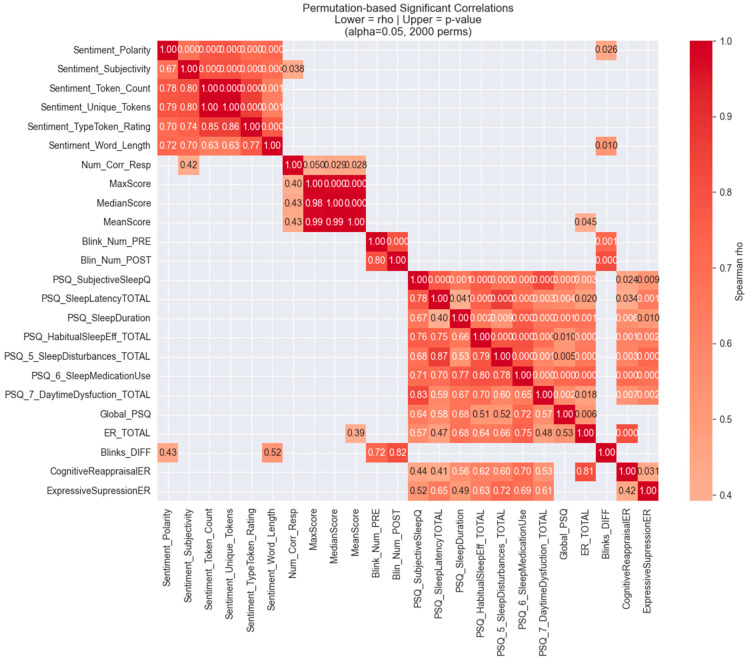
Annotated heatmap displaying significant effects only, with the lower triangle representing correlation coefficients (ρ) and the upper triangle reporting corresponding *p*-values. All Sentiment variables represent different aspects of aesthetic experience as reflected in participants’ Expressive Writing and analyzed through natural language processing; Num_Corr_Resp, MaxScore, MediaScore, and MeanScore are the dependent variables of the WM task, reflecting different aspects of WM capacity; Blink_Num_PRE, Blink_Num_POST, and Blinks_DIFF are the scores from the spontaneous Eye Blink Rate method reflecting DA. All PSQ variables represent different aspects of sleep quality. Global_PSQ = total sleep quality. CognitiveReappraisalER and ExpressiveSupressionER are the two different ER strategies we assessed, and ER-TOTAL is the overall ER score.

Significance was assessed using an alpha level of 0.05. Only correlations surviving this threshold were retained for interpretation, while non-significant values were masked. To facilitate interpretation, we constructed an annotated heatmap (see Figure 3) displaying significant effects only, with the lower triangle representing correlation coefficients (ρ) and the upper triangle reporting corresponding *p*-values. This permutation-based procedure allowed for robust inference without relying on parametric assumptions while also reducing the likelihood of inflated false positives.

The permutation-based Spearman correlations revealed several significant associations across behavioral performance, sentiment features, DA as indicated by sEBR, sleep quality, sentiment indices, and ER measures.

**Hypothesis** **1.** *CREATE platform development: There will be a positive triple association between WM task performance, ER, and creative expression (EW and aesthetic evaluations as measured by sentiment indices).*

Task performance, indicating WM capacity, was positively associated with one sentiment score, as shown by the significant correlation between the number of correct responses and Subjectivity, ρ = 0.42, *p* = 0.028. Also, Emotion Regulation (ER_TOTAL) correlated significantly with WM task performance (mean score: ρ = 0.39, *p* = 0.045).

**Hypothesis** **2.** *The modulatory role of DA: Dopamine activity (as measured by sEBR) will be significantly correlated with WM performance, ER scores, EW indices of verbal creativity and emotionality, and sleep quality measures.*

There were no significant relationships between the sEBR scores and either the ER or the WM task scores.

**Hypothesis** **3.** *The role of sleep quality in cognition and ER: Finally, we expect that sleep quality will be positively associated with both ER and creative expression (EW).*

Overall, ER was significantly associated with all sleep quality variables. More specifically, Emotion Regulation (ER_TOTAL) was significantly related to overall sleep quality (Global PSQI: ρ = 0.53, *p* = 0.006) and all other PSQI subscales: Subjective Sleep Quality (PSQI_SubjectiveSleepQ: ρ = 0.57, *p* = 0.003), Sleep Latency (PSQI_SleepLatencyTOTAL: ρ = 0.47, *p* = 0.020), Sleep Duration (PSQI_SleepDuration: ρ = 0.68, *p* = 0.001), Habitual Sleep Efficiency (PSQI_HabitualSleepEff_TOTAL: ρ = 0.64, *p* < 0.001), Sleep Disturbances (PSQI_5_SleepDisturbances_TOTAL: ρ = 0.66, *p* < 0.001), Sleep Medication Use (PSQI_6_SleepMedicationUse: ρ = 0.75, *p* < 0.001), and Daytime Dysfunction (PSQI_7_DaytimeDysfunction_TOTAL: ρ = 0.48, *p* = 0.018).

Following a similar pattern, the ER strategy of Cognitive Reappraisal was significantly related to all PSQI subscales, though not to overall sleep quality. Specifically, it was significantly related to the following: Subjective Sleep Quality (PSQI_SubjectiveSleepQ: ρ = 0.44, *p* = 0.024), Sleep Latency (PSQI_SleepLatencyTOTAL: ρ = 0.41, *p* = 0.034), Sleep Duration (PSQI_SleepDuration: ρ = 0.56, *p* = 0.006), Habitual Sleep Efficiency (PSQI_HabitualSleepEff_TOTAL: ρ = 0.62, *p* = 0.001), Sleep Disturbances (PSQI_5_SleepDisturbances_TOTAL: ρ = 0.60, *p* = 0.003), Sleep Medication Use (PSQI_6_SleepMedicationUse: ρ = 0.70, *p* < 0.001), and Daytime Dysfunction (PSQI_7_DaytimeDysfunction_TOTAL: ρ = 0.53, *p* = 0.007).

An identical pattern was shown by the ER strategy of Expressive Suppression, which was significantly related to all PSQI subscales, though not to overall sleep quality. Specifically, it was significantly related to the following: Subjective Sleep Quality (PSQI_SubjectiveSleepQ: ρ = 0.52, *p* = 0.009), Sleep Latency (PSQ_SleepLatencyTOTAL: ρ = 0.65, *p* = 0.001), Sleep Duration (PSQI_SleepDuration: ρ = 0.49, *p* = 0.010), Habitual Sleep Efficiency (PSQI_HabitualSleepEff_TOTAL: ρ = 0.63, *p* = 0.002), Sleep Disturbances (PSQI_5_SleepDisturbances_TOTAL: ρ = 0.72, *p* < 0.001), Sleep Medication Use (PSQI_6_SleepMedicationUse: ρ = 0.69, *p* < 0.001), and Daytime Dysfunction (PSQI_7_DaytimeDysfunction_TOTAL: ρ = 0.61, *p* = 0.002).

#### 3.2.2. Intercorrelations

Sentiment Features: Not surprisingly, sentiment indices showed strong intercorrelations. Sentiment Polarity, Subjectivity, Token Count, Unique Tokens, Type–Token Ratio, and Word Length were all significantly associated (ρ = 0.63–0.86, *p* < 0.001).

Task Performance (WM) Measures: As expected, accuracy-related variables (number of correct responses; maximum, median, and mean scores) were highly intercorrelated, ρ = 0.99, *p* < 0.001. The number of successive correct responses also correlated strongly with performance indices (e.g., MaxScore: ρ = 0.83, *p* < 0.001).

Dopamine Activity (as assessed by sEBR): Baseline blinking (Blink_Num_PRE) correlated strongly with post-task blinking (ρ = 0.80, *p* < 0.001). Baseline blinking was also related to Subjective Sleep Quality (ρ = 0.40, *p* = 0.045).

Sleep Quality: As expected, Subjective Sleep Quality was strongly related to all major PSQI domains, including Latency (ρ = 0.78, *p* < 0.001), Duration (ρ = 0.67, *p* = 0.003), Habitual sleep Efficiency (ρ = 0.76, *p* = 0.002), Sleep Disturbances (ρ = 0.68, *p* = 0.010), Medication Use (ρ = 0.77, *p* = 0.001), and Daytime Dysfunction (ρ = 0.83, *p* < 0.001). Global PSQI scores correlated positively with these same components (ρ = 0.53–0.64, all *p* < 0.05).

Emotion Regulation: Emotion Regulation (ER_TOTAL) correlated with Cognitive Reappraisal (ρ = 0.81, *p* < 0.001) but not with Expressive Suppression.

Overall, the results highlight a strong clustering of performance indices; robust interrelations among subjective sleep domains; and meaningful links between WM scores, aesthetic experience variables as indicated by sentiment scores, blinking dynamics, sleep quality, and ER.

##### Group Comparisons

With the previous Permutation Correlations, we tested for an association between subjective sleep quality scores and sEBR, but no significant correlation emerged. However, if a relationship is driven by contrasts between more extreme groups, correlation may fail to detect it. The previous literature suggests that dopamine-related differences may manifest more robustly between individuals with good versus poor sleep quality [73] rather than incrementally across the entire sleep quality spectrum. Therefore, we conducted a group comparison based on sleep quality classification (Good Sleepers vs. Poor Sleepers). Specifically, we separated the participants into two groups (Good Sleepers, N = 9, and Poor Sleepers, N = 18) depending on their sleep quality, as indexed by a PSQI threshold of 5, with a total PSQI score < 5 indicating a Poor Sleeper. Given the small and unequal group sizes, which resulted in violations of normality and the homogeneity of variance assumptions for some variables according to the Shapiro–Wilk and Levene’s tests in our exploratory analyses, we used the non-parametric independent Mann–Whitney U test to assess group differences in all the variables included in the previous analyses. The results indicated that Poor Sleepers (M = 94.50, SD = 66.99) had significantly more sEBR pre than Good Sleepers (M = 33.40, SD = 20.77), *p* = 0.040. However, post-sEBR did not differ significantly among Good Sleepers (M = 41.75, SD = 33.44) and Poor Sleepers (M = 94.55, SD = 53.52), and this was due to the post-sEBR increase manifested by Good Sleepers. This indicates a beneficial task effect in the DA of people with good quality of sleep, further discussed in the Section 4.

#### 3.2.3. Analysis Examining Hypothesis 4: Aesthetic Experience and WM

**Figure 4:**
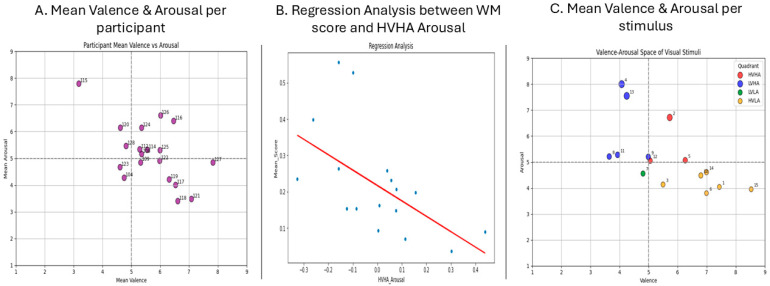
Visualization of the affective ratings in a 2D space per participant (**A**) and per stimulus (**C**). The individual deviations from the mean arousal rating at the group level for HVHA were used as a predictor to estimate the mean WM performance and are visualized in the regression plot (**B**).

Hypothesis 4 suggested the following: given the significant influence of WM in aesthetic experience, we expected that if our WM task indeed taps into WM, and if our visual art stimuli elicit aesthetic experiences, then WM task scores will be related to aesthetic experience scores. For each participant, we computed the mean valence and arousal ratings (Figure 4A). We also computed the mean valence and arousal for each different image (Figure 4B). These values were represented in a 2D space with valence in the horizontal axis and arousal in the vertical axis. Figure 4A,C are divided into four distinct quartiles: (1) high valence and high arousal/HVHA, (2) low valence and high arousal/LVHA, (3) low valence and low arousal/LVLA, and (4) high valence and low arousal/HVLA. This visualization may offer great insight and is very common in affective computing studies [70]. Since arousal is the easiest emotional dimension to identify in neurophysiological recordings and neuropsychological experiments [70], we focused only on the positive and high-arousing stimuli (HVHA), and we computed the signed differences in the individual arousal ratings from the average ones for those stimuli only. We used this variable as a predictor and as a dependent variable for the mean score in the WM task (Figure 4B).

The model explains ~30.8% of the variance in Mean_Score, which suggests a moderate fit for a single-predictor model. The F-statistic (7.131) and its associated *p*-value (0.0168) indicate that the model is statistically significant at the 5% level. When there is no difference between the individual arousal responses for the HVHA arousal stimuli and the group average responses, the expected mean score in the WM task is 0.2171. For each 1-unit increase in the individual arousal rating for the HVHA arousal stimuli, the mean score of the WM task decreases by 0.423 on average, holding all else constant. This negative relationship is statistically significant (*p* = 0.017). The Durbin–Watson value was at 1.57, indicating that the model suffers from mild positive autocorrelation. However, this is not a major concern with cross-sectional data. The Omnibus and Jarque–Bera tests (Omnibus *p* = 0.049) suggest **some non-normality** in residuals. Moreover, the residuals were moderately skewed since the skewness value was slightly larger than unity. A detailed analysis of the model performance is contained in Table 5.

## 4. Discussion

The main aim of the present study was to assess the feasibility of the CREATE platform, as well as the validity of the measures included. Our main feasibility endpoints were recruitment, adherence, and usability. More specifically, we were first interested in the recruitment and adherence rates: recruitment was not very easy, as there were some people (nine of those approached initially) who were not willing to devote half an hour to the experimental procedures. Nevertheless, the vast majority of those approached, and almost everyone who was assessed for eligibility (i.e., 32 of 36), did take part. An important point is that, for reasons of convenience sampling, our participants were mainly cancer-related healthcare professionals, and only one was a cancer survivor, although CREATE is primarily targeted at cancer patients/survivors. That is, from the feasibility results of the present pilot study, we cannot be certain that the same positive pattern of recruitment and adherence would emerge if we approached only cancer survivors or even patients, as they may share difficulties that reflect on their participation, such as cancer-related fatigue. Considering this, the main goal of our second study is to recruit only cancer survivors and patients by first adjusting CREATE to the feedback we received. A longitudinal RCT design, with repeated platform engagement, is also necessary for our future study to rigorously investigate whether usability and adherence change when participants engage with the platform more than once. Nevertheless, the adherence rate for this study was 100%, which is encouraging. Secondly, we assessed the platform’s usability quantitatively, where the participants reported overall high to very high usability rates. We also recorded the participants’ qualitative feedback, as our team is devoted to co-creation, which provided valuable insights into the improvements that can be made for our second study. We suggest that any CCT should follow this method, so as to increase their usability and adherence rates as much as possible.

In terms of validity, given that the CREATE platform is designed to facilitate ER through WM cognitive training, creativity through EW, and aesthetic experience, we formed relevant predictions. As informed by the literature, we predicted that there would be significant positive relationships between WM, ER, and sEBR. We further predict that DA, as measured by sEBR, will be related to sleep quality scores, thus further validating our measures as assessments of functions modulated by DA. We also expected that sleep quality would be related to ER and creativity (EW) scores. Finally, we predicted that WM would be associated with the aesthetic experiences elicited by our art stimuli.

Working Memory is considered the main cognitive modulator of emotional control [18]. This is supported by the very few studies that have shown the effects of WM training in downregulating anxiety [19,20] and depression (see [18] for a review). Therefore, we first expected that performance in the WM computerized game (number of correct trials, RT in correct trials, and maximum number of blocks remembered) would be positively related to scores from the ER scale. Our analyses confirmed this prediction, as the WM mean task score we used was significantly related to the total ER score, indicating that the better the WM capacity, the better the emotional control ability. Given the previously found relationships between WM and ER, our overall WM-ER results not only add to these findings but also underline the validity of our game as a WM task, as if it did not tap into WM effectively, performance on this task would most likely not be correlated to ER.

An additional important result of the current study was that although the poor sleep quality group initially had significantly higher sEBRs than the good sleep quality participants, after executing the WM and the Creativity–Aesthetic Experience tasks, this difference disappeared, and this was due to the increase in post-sEBR manifested in the good sleep quality group. In other words, the participants who slept well enjoyed a DA increase from the WM and Creativity–Aesthetic Experience tasks, as opposed to those who slept more poorly, whose dopamine levels were at a ceiling before the intervention and remained as such after engaging with the platform. The first line of support for this dopamine increase in Good Sleepers comes from studies showing that even a single trial of engaging with a computerized cognitive task can increase DA. For example, a task-switching PET study [74] showed that acute increases in cognitive demand can induce measurable dopamine release. Even though it was not a training intervention over time, it shows that the dopaminergic system is highly plastic in the short term. Another plausible explanation of this finding could be the following: in a recent study in mice, acute sleep deprivation resulted in a short-term dopamine surge [75,76]. It could be that the limited sleep deprivation of our poor sleep group led to such a DA increase, leaving no room for further increase from the CREATE tasks. This, however, is not to claim that people should be deprived of quality sleep to increase their DA, as the dramatic effects of systematic sleep deprivation are now well established [60]. Instead, it does suggest that such a dopamine increase can be accomplished by engaging with the CREATE platform, therefore supporting the benefits of this approach for brain health. However, the longevity of this benefit is yet to be established by a future study testing the CREATE platform’s effects with a longitudinal design.

Conversely, we did not find any associations between sEBR, indicating DA, WM, ER, and sleep quality, although we predicted otherwise. This could be attributed to the very high variability in sEBR, as indicated by the standard deviation of the sEBR, probably masking any underlying relationship with the rest of the scores. However, the sleep group differences in sEBR suggest that when the participants were separated into groups, the previously masked contrasts in sEBR were probably now magnified, hence the significant group differences in sEBR. However, group comparisons are not a direct test of relationships between sEBR and the rest of the measures; therefore, in a future study, a much larger sample must be included to normalize the sEBR variability. Nevertheless, the difference between pre- and post-sEBR was significantly related to two sentiment indices, those of Polarity and AWL. Put differently, the less DA increased after the task, the more positively polarized the words (i.e., positive emotional expression) and the more our participants were linguistically creative. This could suggest that for more creative EW, relatively balanced DA levels are required. Several lines of research support this, with low dopamine levels leading to creative blocks [75] and very high DA probably leading to hyperactive states that can hinder creativity overall [77]. This constitutes very important information for the design of the CREATE platform, which aims at boosting creativity, as it suggests that a balance in DA, rather than a raw increase, is needed.

Overall, these results suggest that our sEBR measure is a rather valid indication of DA, though more testing is needed. In sum, sleep quality does not predict sEBR gradually across the continuum, but poor sleep may push people into a distinct dopaminergic state (high tonic sEBR). Also, Good Sleepers show dynamic dopamine modulation (a baseline low was associated with post-task increase), whereas Poor Sleepers show elevated, rigid dopamine levels (a high baseline was associated with a flat response). Moreover, balanced DA levels are required to boost creativity rather than an increase in DA. Although these findings related to dopaminergic activity are interesting, they cannot be generalized due to single measurements and a small sample size, and they would require a longitudinal follow-up, a larger sample size, and (ideally) a more direct DA measure.

Opposite to what we predicted, however, none of the sleep quality indices were associated with any WM scores. There are several reasons for this null result. Firstly, the PSQI assesses sleep quality over the past month; however, our WM task assesses WM capacity at that moment [78]. Such a timescale mismatch can eliminate the correlations between them. Secondly, as aforementioned, this could be due to the complexity of the relationship between sleep and cognition. Nevertheless, all the PSQI subscales and the total score were significantly related to the total ER score, as well as to the indicators of the Cognitive Reappraisal and the Expressive Suppression ER strategies. This suggests, however, that worse sleep quality (as indicated by higher PSQI scores) is related to more ER, which contradicts the literature discussed earlier. A plausible explanation for this association is that it may reflect a distinction between more use of ER strategies versus more effective ER. That is, it may be that poor sleepers use ER strategies more frequently as compensation for the natural dysregulation effect they experience in response to poor sleep, as opposed to more effectively, and this inflated their ER scores. Overall, apart from partially confirming our third hypothesis and, therefore, the validity of the sleep quality and the ER measures, this finding adds nuance to the existing body of knowledge regarding sleep and ER: rather than poor sleep quality simply impairing ER, it may shift the balance toward greater reliance on deliberate regulation efforts, but these may not necessarily be adaptive or effective.

Another innovative aspect of the CREATE platform is that we tried to gauge creativity through EW (i.e., written emotional evaluations of artistic stimuli). With regard to sleep quality, contrary to what we expected, it was not related to scores reflecting creativity and verbal expression of emotions. There are several reasons why this may have happened. For example, this could be due to a “state–trait” mismatch; that is, the PSQI we employed assessed sleep quality over the past month (i.e., “trait”), whereas our sentiment indices measured verbal creativity and verbal affective expression in each moment only (i.e., “state”). In a future study, the EW task should be repeated several times on different days so that its timescale matches that of the sleep quality measurement. Our null findings could also be due to the specificity of sleep’s effects on cognition. For example, the review in [79] describes several studies showing how sleep loss can have a clear negative effect on executive functions, attention, and memory, but evidence of such an influence on other domains is mixed or weak. Another study supporting the specificity of sleep’s effects on cognition supports the idea that different sleep stages may have a differential contribution to separate aspects of creativity [80]. Recent models also emphasize that creativity emerges from dynamic interactions between directed and undirected attentional pathways [81]. However, our sleep assessment regards overall sleep quality. A future study could employ sleep recordings with polysomnography to investigate such complex relationships between sleep and creativity. Nevertheless, we did find a significant association between WM task accuracy and Subjectivity, suggesting that the higher the WM capacity, the more emotionally expressive the language used in the creativity task. Given the strong bidirectional link between WM and emotional experiences, including emotional expression [82], such a finding was expected. Therefore, we believe that this underlines the validity of both our WM task and the Subjectivity measurement.

The regression analysis showed that higher individual arousal ratings for HVHA stimuli, when compared to the group averages, are significantly associated with a lower mean score in the WM task, which confirms our prediction of a link between WM and aesthetic experience. This underlines the validity of both our WM and our aesthetic experience tasks, as well as the related aesthetic experience metrics. Although the model included only one predictor, it provided a meaningful insight (R^2^ = 0.31) and achieved statistical significance. Although this result highlights the reciprocal relationship between ER and WM capacity, the residual diagnostics indicated mild departures from normality. This is the first time (to the best of our knowledge) that an art evaluation task provided such findings in combination with a computerized cognitive task. Although these results are very encouraging, caution is warranted when interpreting them, as the sample size was limited, which may have increased the probability of a type II error due to decreased statistical power [83]. Another consideration is that we used a cross-sectional design, given that it was a pilot study on the platform’s validity and feasibility; therefore, we cannot suggest any training effects. Even so, we did find several significant results that highlight the validity of the measures and tasks included in the CREATE platform. Our next steps include finalizing the 3-week training phase that has been initiated, as well as enriching CREATE with more CCT and art-related tasks, binaural beats that boost concentration and creativity, videos that elicit aesthetic chills, assessment measures of anxiety and depression levels, and an AI-based agent providing mental health tips tailored to the user’s performance and assessment profile.

Although encouraging, our results should be interpreted with great caution due to the limitations of this study. Firstly, as we aimed to assess the feasibility and validity of the CREATE platform and not its effectiveness, we used an exploratory design, and the trial was not pre-registered. Additionally, the cross-sectional, correlational aspects of our design do not allow for training effect conclusions, and a longitudinal RCT design is required instead. Secondly, the small sample size, typical of pilot studies such as the current one, leaves room for error due to the decreased statistical power [83]. Therefore, our next step is to conduct a longitudinal RCT with a large sample size predetermined by a priori power analysis.

## 5. Conclusions

In conclusion, this study provided the first evidence that the newly developed CREATE platform demonstrates substantial promise as a multi-modal digital intervention. It seeks to enhance ER by integrating WM training, aesthetic engagement, and creative expression through the expression of emotions elicited from famous paintings. It is based on a theoretical model formulated by a theoretical hypothesis claiming a triple interaction between ER, WM, and creativity while attributing a modulatory role to both DA and sleep quality. Based on this model, the system’s technical and functional requirements were co-designed through participatory design workshops with all the contributing institutions.

Overall, the study results were mostly consistent with the theoretical model and validated a positive association between WM performance, ER capacity, and emotional EW, with DA and sleep quality showing at least a partially modulatory role. Therefore, future studies could integrate sleep quality and DA assessment in personalized digital and mental health tools.

These results support the scientific merit of the CREATE platform and its underlying theoretical model. The present study integrates multiple validated dimensions, such as (1) cognitive performance, (2) affective expression, (3) DA, and (4) sleep quality, into a unified, user-friendly, digital environment. CREATE’s multi-faceted architecture sets a new standard for how cognitive health and mental well-being can be simultaneously assessed, trained, and monitored.

The CREATE platform also holds socially transformative potential since it offers a scalable, low-cost, and evidence-based intervention that can be employed to underserved populations with limited access to healthcare services. It also introduces a data-rich, adaptive model for early intervention, which could be used to reduce the financial cost of the healthcare systems for treating chronic emotional disorders. Its evidence-based intervention can be used by policymakers and public health professionals as a promising path to address post-pandemic emotional distress in vulnerable groups such as young adults and patients with chronic illnesses or cancer. The CREATE platform was developed through a modular design and agile methodology, which offers a blueprint for future interdisciplinary studies between cognitive neuroscience, software engineering, artificial intelligence, psycho-informatics, and affective computing.

To sum up, the CREATE platform exemplifies a next-generation, digital mental health tool. It is strongly anchored in affective neuroscience, enriched with art and creativity, and validated through behavioral and biological indicators. The interdisciplinarity of the authorship ensures its deployment in larger, longitudinal trials in which we will investigate its role as an intervention tool and a scientific and societal enabler in the quest for scalable, personalized, and emotionally intelligent mental health care.

## Figures and Tables

**Table 1:** Description of the WM parameters used in the CREATE memory game.

Game Parameters	Type	Description
Num_Correct_Responses	int	Total number of times the player **responded with the correct full sequence** (i.e., all buttons clicked in correct order).
Num_Total_Responses	int	Total number of **attempted responses**, regardless of correctness. Used as the denominator in score calculations.
Max_Score	double	The **maximum score** (correct/total) achieved at any point during the session or across trials/levels. Represents peak performance.
Median_Score	double	The **middle value** of all recorded scores across trials (not the average). Less sensitive to outliers and gives a robust estimate of typical performance.
Mean_Score	double	The **average score** across all trials. Calculated as the sum of per-trial scores divided by the number of trials. Reflects overall accuracy.
Count_Above_0.25	int	The number of trials in which the **score was greater than 0.25**. It indicates how frequently the user performed above a low-to-moderate threshold.
Count_Above_0.5		The number of trials in which the **score was greater than 0.5**. Indicates how often the user performed above a moderate-to-high accuracy threshold.
Successive_Correct_Count	int	The **longest streak** of consecutive correct responses (i.e., in how many back-to-back rounds did the user achieve the correct sequence). A measure of consistency.

**Table 2:** Descriptive statistics of all Affective Usability and User Experience subscales.

	Mean	Std. Deviation	Max Subscale Likert Score
Computer Literacy Total	3.40	0.24	4
Affective Evaluation Total	5.54	0.55	7
Usability Total	4.70	0.29	5
Interface Total	1.70	0.35	2
Perceived Beneficence	3.70	0.68	5
User Engagement Total	3.58	0.51	5
Sustainability Total	6.60	1.35	8

**Table 3:** Main themes and subthemes form the Thematic Analysis of the qualitative data.

Main Theme	Subthemes
1. User Engagement	Interactivity
	Mind-provoking
	User-friendly
2. Technology’s contribution to training	Gamified nature of training
	Overall integration of technology
3. Cognitive/affective states elicited	Motivating
	Challenging
	Creative
	Fun
4. Drawbacks	Functionality (technical issues)
	Design of environment
	Level of difficulty

**Table 4:** Descriptive statistics (means and SDs) for all variables included in the analysis.

		Mean	SD
**WM task performance**	Num_Corr_Resp	17.52	10.03
	WM_max	0.39	0.19
	WM_median	0.27	0.19
	WM_mean	0.25	0.16
	Count_Above_0.5	6	10.55
**Emotion Regulation Questionnaire**	ER_Cog_Reapp	29.82	5.05
	ER_Expr_Supr	12.18	5.81
	ER_Total	42	6.88
**Pittsburgh Sleep Quality Index scores**	PSQI_Subj_Sleep_Q	1.32	0.65
	PSQI_Sleep_Latency	1.32	1.04
	PSQI_Sleep_Duration	1.32	0.72
	PSQI_Sleep_Eff	0.23	0.53
	PSQI_Sleep_Distur	1.14	0.47
	PSQI_Daytime_Dysf	1.18	0.73
	Global_PSQI	6.60	2.26
**sEBR before training (baseline)**	sEBR_Pre	71.26	56.78
**sEBR after training**	sEBR_Post	77.77	52.94
**Difference (sEBR Post–sEBR_Pre)**	sEBR_Diff	4.94	32.52
**Sentiment analysis**	**Polarity**	0.068	0.09
	**Subjectivity**	0.488	0.14
	**Word Length**	6.288	0.59
	**Token Count**	10.06	5.25
	**Unique Tokens**	9.44	4.71
	**Type-Token Ratio**	0.96	0.34

**Table 5:** Regression analysis model performance report.

Component	Metric	Value	Interpretation
Model Fit	R-squared	0.308	~30.8% of variance in Mean_Score is explained by HVHA_Arousal.
	Adjusted R-squared	0.265	Adjusts R^2^ for number of predictors; still moderate model fit.
	F-statistic	7.131	The overall model is statistically significant.
	Prob (F-statistic)	0.017	*p* < 0.05 confirms model significance.
	AIC (Akaike Information Criterion)	−22.22	Lower is better: AIC is used for model comparison.
	BIC (Bayesian Information Criterion)	−20.44	Lower is better: Penalizes for model complexity.
Predictor Performance	Intercept (const)	0.2171	Expected value of Mean_Score when HVHA_Arousal = 0.
	Std. Error (Intercept)	0.029	Low standard error, indicates precision.
	*p*-value (Intercept)	<0.001	Highly significant.
	HVHA_Arousal Coefficient	−0.423	Negative relationship: higher arousal leads to lower Mean_Score.
	Std. Error (HVHA_Arousal)	0.159	Moderate uncertainty around coefficient.
	t-statistic (HVHA_Arousal)	−2.670	Coefficient is significantly different from zero.
	*p*-value (HVHA_Arousal)	0.017	Statistically significant (*p* < 0.05)
	95% Confidence Interval	[−0.759, −0.087]	There is 95% confidence that the true coefficient lies in this range.
Residual Diagnostics	Durbin–Watson	1.568	Slight positive autocorrelation; not concerning for cross-sectional data.
	Omnibus Test	6.035	Primary normality test for residuals.
	Prob (Omnibus)	0.049	Mild evidence against normality.
	Jarque–Bera	3.795	Secondary normality check.
	Skewness	1.096	Residuals are moderately right-skewed.
	Kurtosis	3.509	Close to normal (ideal = 3), mild leptokurtic shape.
Multicollinearity Check	Condition Number	5.430	Low value (<10.0), so no multicollinearity problems detected.

## Data Availability

The data presented in this study are available upon request from the corresponding author due to ongoing analyses.

## Abbreviations

CCT	Computerized Cognitive Training
DA	Dopamine Activity
ER	Emotion Regulation
EW	Expressive Writing
sEBR	Spontaneous Eye Blink Rate
WM	Working Memory
